# Pol5 is required for recycling of small subunit biogenesis factors and for formation of the peptide exit tunnel of the large ribosomal subunit

**DOI:** 10.1093/nar/gkz1079

**Published:** 2019-11-20

**Authors:** Christina M Braun, Philipp Hackert, Catharina E Schmid, Markus T Bohnsack, Katherine E Bohnsack, Jorge Perez-Fernandez

**Affiliations:** 1 Department of Biochemistry III, University of Regensburg, Universitätstrasse 31, 93053 Regensburg, Germany; 2 Department of Molecular Biology, University Medical Center Göttingen, Humboldtallee 23, 37073 Göttingen, Germany; 3 Göttingen Center for Molecular Biosciences, Georg-August University, Göttingen, Justus-von-Liebig-Weg 11, 37077 Göttingen, Germany

## Abstract

More than 200 assembly factors (AFs) are required for the production of ribosomes in yeast. The stepwise association and dissociation of these AFs with the pre-ribosomal subunits occurs in a hierarchical manner to ensure correct maturation of the pre-rRNAs and assembly of the ribosomal proteins. Although decades of research have provided a wealth of insights into the functions of many AFs, others remain poorly characterized. Pol5 was initially classified with B-type DNA polymerases, however, several lines of evidence indicate the involvement of this protein in ribosome assembly. Here, we show that depletion of Pol5 affects the processing of pre-rRNAs destined for the both the large and small subunits. Furthermore, we identify binding sites for Pol5 in the 5′ external transcribed spacer and within domain III of the 25S rRNA sequence. Consistent with this, we reveal that Pol5 is required for recruitment of ribosomal proteins that form the polypeptide exit tunnel in the LSU and that depletion of Pol5 impairs the release of 5′ ETS fragments from early pre-40S particles. The dual functions of Pol5 in 60S assembly and recycling of pre-40S AFs suggest that this factor could contribute to ensuring the stoichiometric production of ribosomal subunits.

## INTRODUCTION

**Figure 1.**
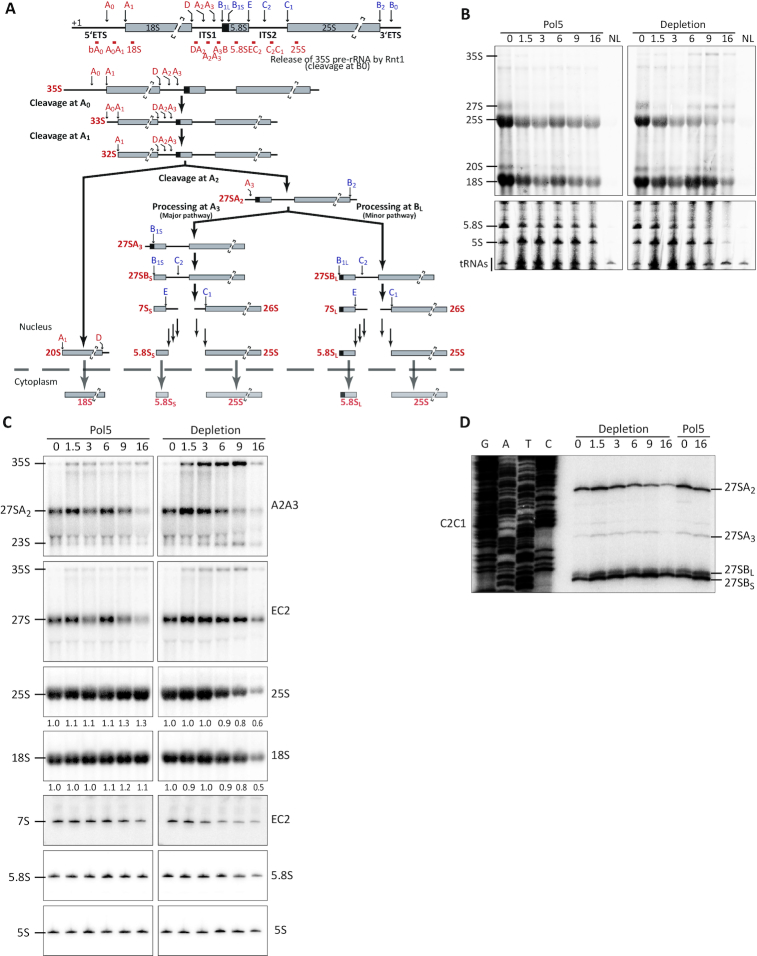
Depletion of Pol5 impairs pre-rRNA processing at the A2 and C2 cleavage sites. (**A**) Schematic representation of pre-rRNA processing in *S. cerevisiae*. Positions of the probes used for northern blot analysis and primer extension are indicated and named according to the region where they hybridize. (**B**) Northern blot analysis of RNAs produced during a metabolic labeling experiment. Cells expressing Pol5 from a plasmid (Pol5) or containing an empty plasmid backbone (Depletion) were cultivated in glucose-containing medium for the indicated times (hours) to allow depletion of the endogenously expressed Pol5. At the indicated times, cells were treated for 20 minutes with 4tU. Total RNA was extracted, resolved by gel electrophoresis in denaturing agarose (upper panels) or acrylamide gels (lower panels) and transferred to a nylon membrane. To detect labeled RNAs, membranes were processed as indicated in methods. NL indicates RNAs derived from cells not treated with 4tU. (**C**) Northern blot analysis of RNAs with radioactively labeled probes. Total RNA obtained at indicated depletion times (in hours) was resolved in denaturing agarose (top four panels) or acrylamide (bottom three panels) gels and transferred to a nylon membrane, which was hybridized with the indicated oligonucleotide probes. (**D**) Primer extension analysis of RNAs obtained during Pol5 depletion. Total RNAs obtained at the indicated depletion times (hours) were used in primer extension assay with the indicated oligonucleotides. Reaction products were separated by denaturing polyacrylamide gel electrophoresis and radioactively labeled cDNA fragments were detected. Sequencing reactions on a plasmid (K375) encoding a full ribosomal DNA copy were performed in parallel (lanes G, A, T and C).

The synthesis of ribosomes is an essential cellular process that enables the production of all proteins. In *Saccharomyces cerevisiae*, hereafter called yeast, more than 200 assembly factors (AFs) participate in the correct formation of ribosomes. AFs are required for processing and folding of the ribosomal RNA (rRNA) as well as for the incorporation of ribosomal proteins (RPs) (1). The 35S pre-rRNA is synthesized by RNA polymerase I, and it contains the 18S, 5.8S and 25S rRNA sequences, which are separated by two internal transcribed sequences (ITS1 and ITS2) and flanked at both ends by the external transcribed sequences (ETS1 and ETS2) (Figure 1A). Early pre-ribosome assembly events, which include assembly of some ribosomal proteins, the initial pre-rRNA cleavages that separate the biogenesis pathways of the small and large ribosomal subunits (SSU and LSU respectively), and many rRNA modifications, can occur co-transcriptionally (2,3).

During the early steps of ribosome assembly, a large number of AFs associate with the nascent transcript to form the SSU processome (3,4). These early assembly factors were named as **U t**hree **p**roteins (Utp) because of their association with the U3 small nucleolar (sno)RNA. Formation of the SSU processome is a hierarchical process initiated by seven AFs called t-Utps due to their putative role in rDNA **t**ranscription (5,6). The t-Utps assemble to form the t-UTP complex independently of ongoing ribosome biogenesis (7) and associate with the 5′ ETS of the pre-rRNA (8,9). Association of the t-UTP complex with the pre-rRNA transcript allows recruitment of the UTP-B complex, the U3 snoRNP and Rrp5, which subsequently facilitate the association of other, downstream AFs (6,10–12). Formation of the SSU processome is required for pre-rRNA cleavages at sites A0, A1 and A2 that will allow removal of the ETS1 region and separate the pre-rRNAs of the SSU and LSU (13–15). The precursor of the SSU (pre-40S), containing the 20S pre-rRNA, is rapidly exported to the cytoplasm (16). The final maturation steps of the SSU include a translation-like cycle to proofread the pre-40S particle and achieve a competent state for the final cleavage at the 3′ end of the 18S rRNA (17–19).

In parallel, initial maturation of the large subunit requires the hierarchical association of the A_3_ AFs, which are required for trimming of the 5′ end of the 5.8S (13,20,21). Afterwards, the association of several factors prepares the 27SB precursor for cleavage in ITS2 (at site C2; Figure 1A) that produces the 7S and 26S pre-RNAs that include the 5.8S and 25S rRNA sequences respectively (22–24). Before cleavage in ITS2 occurs, the 5S rRNA, produced by RNA polymerase III, is incorporated as a ribonucleoprotein particle containing the RPs L5 and L11 (uL18 and uL5 respectively) (25). However, a significant conformational rearrangement that occurs during the late nuclear steps of pre-60S biogenesis is required to form the mature central protuberance structure (26,27). The 3′-5′ exonuclease complex, the exosome, trims the 3′ end of the 7S pre-rRNA to produce the 5.8S rRNA (28–30) and the 5′-3′ exonucleases Rrp17 and Rat1-Rai1 remove the 5′ end of the 26S pre-rRNA to produce the 25S rRNA (31,32). During the late nuclear stages, the pre-60S particle acquires competence for transport through nuclear pores by the association and dissociation of several AFs (22,33).

Despite its initial characterization as a B-type DNA polymerase, Pol5 is functionally unrelated to these enzymes (34,35). Instead, Pol5, and its homolog in humans MYBBP1a, belong to a family of predicted transcription regulators and both appear to play roles in ribosome biogenesis (34,36,37). However, the precise function(s) of Pol5 remains unclear. Interestingly, Pol5, together with six t-Utps, were identified as a protein complex called UTP-A by the group of Greenblatt (38), suggesting that Pol5 may participate in early events during ribosome assembly. In this work, we provide evidence that Pol5 participates in the maturation of both ribosomal subunits. During pre-60S biogenesis, Pol5 associates with domain III, where it facilitates the stable association of ribosomal proteins that form the outer face of the peptide exit tunnel (L19 (eL19), L25 (eL25), L27 (eL27) and others). This event is a critical pre-requisite for C2 cleavage in ITS2 and downstream assembly steps (22,39–41). Moreover, our data indicate the requirement for Pol5 for turnover of excised fragments of the 5′ ETS and recycling of associated SSU AFs. Taken together, these data suggest that Pol5 may contribute to regulating the balance between SSU and LSU production, which is important for maintaining protein homeostasis in growing cells.

## MATERIALS AND METHODS

### Yeast strains and microbiological procedures

Oligonucleotides, plasmids and yeast strains used in this work are listed in Supplementary Tables S1-S3 respectively. Modified yeast strains (Supplementary Table S3) were obtained by homologous recombination using PCR-amplified cassettes. For conditional expression of *POL5*, the *KANMX*::*GAL*::*HA* cassette was inserted immediately upstream of the *POL5* locus as described (42). Genes were C-terminally TAP-tagged by homologous recombination of PCR products obtained from the plasmids pBS1539 (K97) or pYM15-TAP-*URA3* (p96) with the oligonucleotides listed in Supplementary Table S1. Yeast cells were cultured in YPG (1% yeast extract, 2% bacto peptone, and 2% galactose) or YPD (1% yeast extract, 2% bacto peptone and 2% glucose). Minimal media (SC), containing either 2% galactose (SCG) or 2% glucose (SCD), was used for continuous selection of cells containing a plasmid. SC medium lacking uracil was used for 4-thiouracil (4tU) metabolic labeling.

### DNA cloning

The coding sequence of *POL5* was amplified from genomic DNA of the *S. cerevisiae* strain BY4741 (Euroscarf) using oligonucleotides o86 and o87. The PCR product was cloned into the *BamHI* and *NotI* restriction sites of the yeast plasmid pCM182-LEU2-FLAG to obtain ptCMS2.

### Cell lysis for affinity purification

Cell pellets derived from 500 ml exponentially growing cultures of yeast strains expressing TAP-tagged proteins were resuspended in 10 ml Buffer P1 (150 mM KAc, 20 mM Tris, pH 8.0, 5 mM MgCl_2_, 1 mM DTT, 0.2% (w/v) Triton) supplemented with Protease Inhibitors and RNasin (Promega). Mechanical cell lysis was performed at 4°C for 6 × 30 s at 6000 rpm with 5 × 30 seconds pausing in a Precellys Evolution coupled to Cryolys (Bertin Instruments). Cell debris were pelleted by centrifugation at 18 000 g for 15 min at 4°C and clarified lysates were used for western blot analysis and for affinity purification.

### Affinity purification using IgG-coupled beads

Affinity purification was performed as described (10). Elutes were either used for western blotting and Coomassie staining analysis as described below or further processed for downstream RNA analysis (sepharose beads) or semi-quantitative mass spectrometric (qMS) analysis (magnetic beads) as previously described (43), using equal amounts of total protein. Data were normalized by setting the iTRAQ ratios of the bait proteins to one.

### SDS-PAGE and western blotting

Samples for analysis by SDS-PAGE were mixed with 1× Laemmli Buffer and processed as described (44). Approximately 0.2% of whole cell lysate (WCL) and 10% of eluates were loaded. Detection of proteins was performed with antibodies described in Supplementary Table S4 using the chemiluminescence western blotting reagent (Roche) in a LAS-3000 device (Fujifilm).

### RNA extraction and northern blotting

RNAs were extracted using hot acidic phenol/chloroform treatment (45). Approximately 0.15% of WCL and 15% of eluates were analyzed. Northern blotting analysis after RNA separation on denaturing (urea) polyacrylamide gels (for short RNAs) or formaldehyde/MOPS agarose gels (long RNAs) were carried out as described (44). Hybridization with ^32^P-labeled probes (listed in Supplementary Table S1) was performed as previously described (6).

### 4tU metabolic labeling of nascent RNAs

After different depletion periods of *POL5*, 20 AUs of cells were collected from exponentially growing cultures. For pulse labeling, 200 μM 4tU was added and cultures were incubated for an additional 20 min at 30°C. A non-labeled control was performed using 20 AUs of exponentially growing cells but without addition of 4tU. The concentration of 4tU used causes only a mild change (10–15%) in the doubling time when cells were cultivated for 24 h in presence of 4tU (data available upon request). After RNA extraction, 50 μg total RNA were processed as previously described (46,47). Total RNA was precipitated and resuspended in 20 μl H_2_O. 5 μl of each sample were separated on denaturing (formaldehyde), MOPS agarose and (urea), TBE polyacrylamide gels. Membranes were treated as previously described (46,47) with IR-dye conjugated Streptavidin (1:10,000; IRDye 800CW Streptavidin, Pierce) and labeled RNAs were detected using a LI-COR Odyssey imaging platform.

### Primer extension on total RNA

Primer extension analyses were performed as described (48) using ^32^P-labeled primers listed in Supplementary Table S1. Sequencing reactions using the same primers were performed with the sequencing kit from (Thermo sequenase 785001KT Affymetrix). The products of primer extension and sequencing reactions were resolved on denaturing (urea), taurine polyacrylamide gels according to the manufacturer's instructions. Dried gels were exposed to Phosphorimager screens (Fujifilm) and radioactive cDNAs were detected using a FLA-9500 Phosphorimager (Fujifilm). Obtained data were analyzed and further processed in MultiGauge v.3.0.

### Crosslinking and analysis of cDNA (CRAC)

Exponentially growing cells of a yeast strain expressing C-terminally His_6_-TEV protease cleavage site-ProteinA-tagged Pol5 from its genomic locus were crosslinked using UV light at 254 nm before harvesting. Cells were lysed in TMN150 (50 mM Tris–HCl pH 7.8, 150 mM NaCl 1.5 mM MgCl_2_, 0.1% NP-40 and 5 mM β-mercaptoethanol) and RNA-protein complexes were isolated by tandem affinity purification via the ProteinA tag (native) and His tag (denaturing, 6 M guanidium). A partial RNase digest was performed using RNace-IT (Agilent), and the RNA fragments were 5′ ^32^P-labeled and sequencing adaptors were ligated to the 5′ and 3′ ends. Complexes were separated by denaturing polyacrylamide gel electrophoresis, transferred to a nitrocellulose membrane and the region of the gel containing Pol5-associated RNA fragments was excised. RNAs were isolated and reverse transcribed using SuperScript III reverse transcriptase (ThermoFisherScientific). After PCR amplification, the obtained cDNA library was subjected to Illumina sequencing. After removal of barcodes and collapsing of identical sequences, the obtained sequencing reads were trimmed and quality controlled using Flexbar (49), then mapped to the *S. cerevisiae* genome (S228C) using Bowtie 2 (version 2.2.4; (50)). The proportions of reads mapping to different types of RNA were determined using pyCRAC read counting (51) and self-written python scripts were used to map the data onto the available secondary (52) and tertiary (53) (PDB: 5TZS) structures of the rRNA (54).

## RESULTS

### Pol5 is required for the maturation of both ribosomal subunits

To characterize the role of Pol5 during ribosome biogenesis, we first analyzed the effects of Pol5 depletion on cell growth. As Pol5 is an essential protein, *POL5* was expressed under the control of a galactose inducible and glucose repressible GAL1 promoter. Expression of Pol5 from the *GAL* promoter did not cause a substantial change in cell growth when cells were grown in media containing galactose (generation time of wild type strain, 162 ± 9 min and *GAL::HA-POL5*, 176 ± 5 min) and analysis of pre-rRNA intermediates in the these strains by northern blotting did not reveal any significant differences (Supplementary Figure S1A). These results confirm that any alterations in the expression level of Pol5 caused by the promoter exchange do not perturb cell proliferation or ribosome assembly. To enable the function of Pol5 to be analyzed independently of the physiological state of cells due to growth on different carbon sources, cells conditionally expressing Pol5 were transformed with either a plasmid encoding a FLAG-tagged *POL5* (Pol5) or a plasmid backbone (Depletion). Cells were cultivated in glucose-containing medium for 16 h, maintaining exponential growth conditions by diluting the cultures in fresh medium every 6 h. Depletion of the chromosomally encoded Pol5 did not cause any growth defect when an extra copy of *POL5* was provided in the plasmid (Supplementary Figure S1B) implying that expression of *POL5* from an exogenous plasmid supports cell growth. In contrast, growth of the *GAL::HA-POL5* strain carrying an empty plasmid (Pol5 depletion) showed a growth defect detectable after 9 h of culturing in glucose, confirming the importance of Pol5 for cell proliferation (Supplementary Figure S1C). Importantly, western blotting revealed that in this strain, HA-tagged Pol5 was still present 3 h after culturing in glucose and only became undetectable after 6 h of growth in restrictive conditions (Supplementary Figure S1D).

Next, to analyze the requirement for Pol5 for the production of the mature rRNAs, newly synthesized RNAs were metabolically labeled with 4tU after different times of culturing in glucose-containing media. While no changes were observed in the relative levels of the rRNAs in cells complemented with the plasmid expressing *POL5*, the absence of an extra copy of *POL5* lead to decreased synthesis of 25S and 5.8S after 6–9 h of culturing in glucose (Figure 1B, compare ‘Pol5’ and ‘Depletion’ panels). In addition, accumulation of RNAs that likely correspond to the 35S and 27S pre-rRNA species were observed only in the absence of Pol5 after a similar time of culturing in glucose. Moreover, synthesis of the 18S rRNA was affected after 9 h of Pol5 depletion but synthesis of 5S rRNA was only impaired at even longer depletion times. As a complementary approach, nascent RNAs in cells that had been grown in glucose for either 3 or 7 h to deplete endogenous Pol5 were pulse-labeled with [^3^H]-labeled uracil followed by addition of an excess of non-labeled uracil. Analysis of the (pre-)rRNAs present at different chase times showed that after 3 h growth in glucose there was little or no difference between cells expressing or lacking Pol5 (Supplementary Figure S1E). In contrast, after 7 h growth in glucose, cells lacking Pol5 showed impaired production of the mature 25S and 5.8S rRNAs as well as accumulation of the 35S pre-rRNA compared to cells expressing Pol5. In addition, lack of Pol5 caused a shift in the population of 27S pre-RNAs toward the shorter 27SB species, which clearly accumulated over time, as well as causing a strong decrease in the synthesis of the 20S pre-rRNA (Supplementary Figure S1E). Consistent with the metabolic labeling experiments, sucrose gradient analysis of cell extracts showed subunit imbalance and accumulation of half-mer polysomes when Pol5 was depleted (Supplementary Figure S1F and G), further supporting the importance of Pol5 for production of 60S subunits.

Analysis of steady-state levels of pre-RNAs was then performed by northern blotting using specific probes; while the pre-rRNA probes used detect the 35S initial pre-rRNA transcript, they also enable the diverse 27S pre-rRNA species to be differentiated (A2A3 – 27SA2, A3B – 27SA2+27SA3 and EC2 – 27SA2 + 27SA3 + 27SB_S/L_). Increased production of 27SA2 was observed after 1.5 h of growth in glucose in both the strain expressing Pol5 from the plasmid and that lacking Pol5. This likely reflects the higher production of ribosomes after addition of glucose resulting in a switch from A3 cleavage to A2 cleavage (55). After longer depletion of Pol5 (from 6 h), accumulation of the 35S and 23S pre-rRNAs was observed, as well as a mild decrease in the overall levels of the 27S pre-rRNAs (Figure 1C, A2A3 and EC2 probes and Supplementary Figure S2A, A3B probe). However, the observed depletion of 27S pre-rRNA when Pol5 was not expressed predominantly reflects decreased amounts of the early 27SA2 pre-rRNA (a 10-fold decrease of the 27SA2/3 pre-rRNAs compared to a 2-fold decrease in total 27S pre-rRNA species combined) (Figure 1C, A2A3 and EC2 probes and Supplementary Figure S2A, A3B probe). This result also indicates that the 27SB pre-rRNA is either stabilized or mildly accumulates when Pol5 is lacking. The increased levels of the 35S and 23S pre-rRNAs, concomitant with depletion of the 27SA pre-rRNAs indicates impaired cleavage at the A0, A1 and A2 sites. Interestingly, while the 27SB pre-rRNA was stabilized by depletion of Pol5, the level of the 7S pre-rRNA was reduced up to 5-fold, indicating impaired cleavage at the C2 site. Together, these results are consistent with the requirement for Pol5 for production of rRNAs destined for both the SSU and LSU observed in the metabolic labeling experiment. Northern blotting for the mature rRNAs also confirmed the specific depletion of the 25S, 5.8S, and 18S rRNAs after 9 h of culturing in glucose (Figure 1C). Importantly, the levels of other RNAs (e.g. the snoRNAs U3 and U14) remained unaffected by Pol5 depletion and exogenous expression of Pol5 was able to rescue all the observed pre-rRNA processing defects (Figure 1C and Supplementary Figure S2A). Finally, to characterize in more detail the proportion of each 27S pre-rRNA species present in cells lacking Pol5, primer extension was performed on pre-RNAs obtained after different times of culturing in glucose. The results confirmed the predominant depletion of 27SA2, rather than the 27SB pre-rRNA in the absence of Pol5 (Figure 1D). In addition, we did not observe major changes in the ratio 27SB_S_/27SB_L_, indicating that 5′ processing of 27S pre-rRNAs is not affected. Although the 25.5S/26S pre-rRNA species generated by C2 cleavage in ITS2 is a very transient intermediate, a weak signal corresponding to this pre-rRNA was detectable in cells expressing Pol5 and those lacking Pol5 up to 1.5 h of growth in glucose. After prolonged growth in glucose, this signal was no longer visible, consistent with the northern blot analyses indicating that the 7S pre-rRNA, and C2 cleavage, is affected by lack of Pol5 (Supplementary Figure S2B). Taken together, these results demonstrate that Pol5 is an SSU and LSU assembly factor that is required for pre-rRNA processing at the A0, A1, A2 and C2 sites in the 5′ ETS, ITS1 and ITS2.

### Pol5 contacts domain III of the 25S sequence, ITS2, and the 5′ ETS of the pre-rRNA

**Figure 2.**
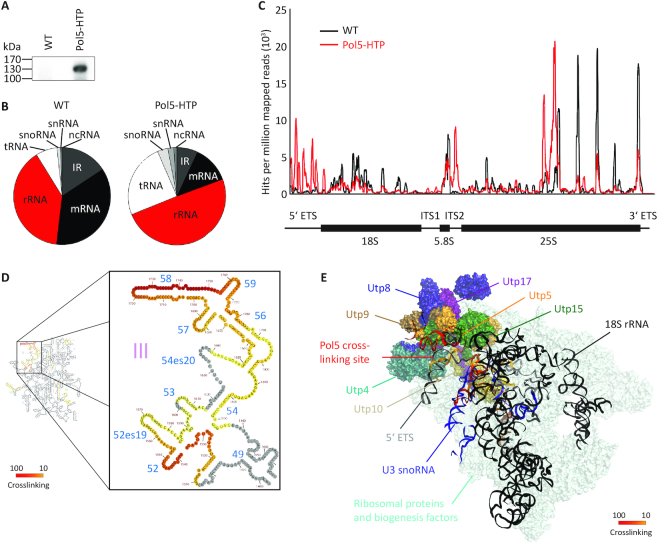
Pol5 crosslinks to the 5′ ETS, ITS2 and domain III of the 25S rRNA sequence. (**A**) Wild-type yeast or yeast cells expressing C-terminally HTP tagged Pol5 were crosslinked *in culturo* using UV light. RNA-protein complexes were affinity purified, and RNAs were trimmed, 5′ labeled using ^32^P and ligated to adaptors. Complexes were separated by denaturing polyacrylamide gel electrophoresis, transferred to a nylon membrane and visualized by autoradiography. (**B**) RNAs isolated from the membrane shown in (A) were reverse transcribed, PCR amplified and subjected to next-generation sequencing. The obtained sequence reads were mapped to the *S. cerevisiae* genome and the relative proportions of reads mapping to genes encoding different types of RNAs is shown. rRNA – ribosomal RNA, tRNA – transfer RNA, snoRNA – small nucleolar RNA, snRNA – small nuclear RNA, ncRNA – non-coding RNA, IR – intergenic region, mRNA – messenger RNA. (**C**) The normalized numbers of reads mapping to each nucleotide of the *RDN37* locus encoding the 35S pre-rRNA is shown above a schematic view of the transcript. (**D**) The number of sequencing reads in the Pol5-HTP dataset mapping to each nucleotide of the 25S rRNA sequence is shown on the secondary structure of the mature 25S rRNA using a color code in which the maximum number of reads is shown in red and lower numbers of reads are shown in yellow. (**E**) The number of sequencing reads in the Pol5-HTP dataset mapping to each nucleotide of the 5′ ETS sequence was mapped onto the tertiary structure of the SSU processome purified via Utp1 and Kre33 (PDB: 5TZS) using a color code as in (D). The t-UTP proteins are highlighted in different colors and labeled. The rRNA sequences and the U3 snoRNA are shown as ribbons in grey and blue respectively, and overlay the RPs and assembly factors, which are shown in pale cyan.

To identify the putative binding site(s) of Pol5 on pre-rRNAs, cross-linking and analysis of cDNA (CRAC) was performed (56). His-TEV protease cleavage site-ProteinA (HTP)-tagged Pol5 was crosslinked to associated RNAs *in culturo* using UV light at 254 nm. After tandem affinity purification of Pol5-containing complexes under native and denaturing conditions, and RNase trimming to leave a specific footprint of Pol5 on its target RNAs, RNA fragments were radiolabeled, and crosslinked complexes were separated by denaturing PAGE. This demonstrated the specific co-purification of RNA fragments with Pol5 (Figure 2A), confirming its association with cellular RNAs. The region of the gel containing these fragments was excised and isolated RNAs were reverse transcribed to generate a cDNA library that was subjected to Illumina sequencing. Consistent with the identification of Pol5 as a ribosome AF, mapping of the obtained sequence reads to the yeast genome showed an increase in the proportion of reads derived from rRNAs in the Pol5 dataset compared to the wild-type control (Figure 2B). Analysis of the distribution of sequence reads mapping to the *RDN37* locus that encodes the 35S pre-rRNA transcript revealed the co-purification of specific pre-rRNA sequences with Pol5, but not the wild-type control (Figure 2C). The most prominent contact sites were found within domain III of the 25S rRNA sequence in helix h58 and helix h52 (Figure 2D). Notably, while these two helices are in close proximity in later pre-60S particles purified via Rix1-TAP and Rpf2-Flag (PDB: 6ELZ, state E; Supplementary Figure S3) they do not orientate toward each other (41). However, during the earlier stages of pre-60S maturation when Pol5 likely acts, this region of the particle is highly flexible (41), suggesting that they likely reflect a single Pol5 binding site. In addition, Pol5 contacts ITS2. As Pol5 is required for cleavage at the C2 site in ITS2 (Figure 1B), our results suggest that Pol5 could connect folding of domain III with the processing of ITS2, as has been described for other AFs and ribosomal proteins (39,45,57–59). Notably, we also detected crosslinking sites of Pol5 in the 5′ ETS (Figure 2C) that cluster at the binding site of the t-UTP complex proteins (Figure 2E). Together, these data demonstrate that Pol5 associates directly with pre-rRNAs and further support roles for Pol5 in maturation of both the small and large ribosomal subunits.

### Pol5 predominantly associates with the 27SB pre-rRNAs

**Figure 3.**
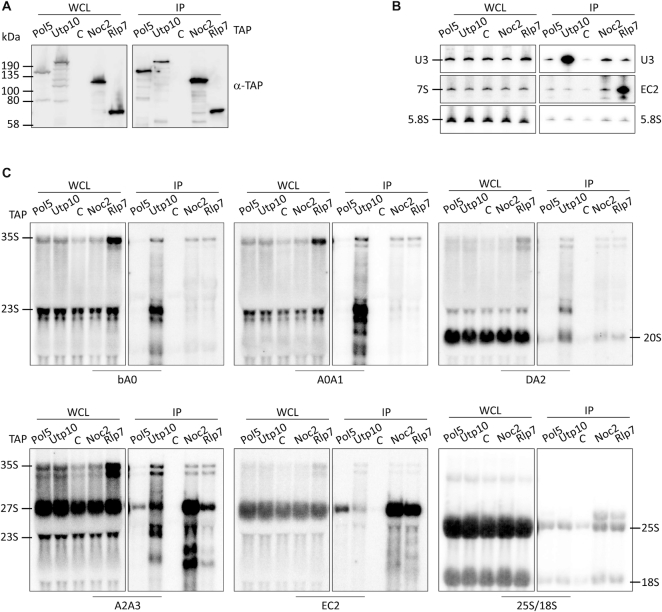
Association of Pol5 and other AFs with pre-rRNAs. (**A**) Pre-ribosomal complexes were isolated via TAP-tagged assembly factors. Whole cell lysates (WCL) and affinity purification samples (IP) were analyzed by western blotting with the indicated antibodies. TAP-tagged proteins are indicated and C indicates the non-tag-containing strain (BY4741) used as a control. (**B**, **C**) Northern blot analyses of pre-rRNAs co-purified with AFs. RNAs enriched in the affinity purifications were extracted and resolved in denaturing acrylamide (B) or agarose (C) gels, transferred to nylon membrane, and hybridized with the indicated, radiolabeled oligonucleotide probes.

To analyze the timing of the association of Pol5 with pre-ribosomal complexes, we TAP-tagged Pol5 and several AFs to compare their associated pre-rRNAs (Figure 3A). As Pol5 is required for cleavage at the C2 site, the AFs Rlp7 and Noc2, which are reported to co-purify pre-60S particles, were selected. Although both AFs associate with pre-ribosomal particles containing 27S pre-rRNAs, Rlp7, but not Noc2, remains associated after C2-site cleavage. In addition, the SSU processome component Utp10 was included as a negative control for association with LSU pre-rRNAs, and a wild-type strain (BY4741) was employed as a control for the unspecific association of pre-rRNAs during the affinity purification. As expected, Utp10 associated mainly with RNA components of pre-40S subunits (the U3 snoRNA, the 35S pre-rRNA, the 23S pre-rRNA and the 20S pre-rRNA) (Figure 3B and C) (5). In contrast, Noc2 and Rlp7 associated with the 27S pre-rRNAs (Figure 3B and C). While both AFs co-purified with the 27SA2 and 27SB pre-rRNAs, Noc2 associated preferentially with 27SA2 and Rlp7 with 27SB (Figure 3C) (60,61). As expected, Rlp7, but not Noc2, remained associated with 7S pre-rRNAs (Figure 3B) (60), consistent with an earlier association of Noc2 with pre-rRNA and a later disassembly of Rlp7. In the case of Pol5, a significant accumulation of the 27SA2 pre-rRNA was observed compared with the untagged control, but the signal was comparable to that observed in the Utp10 purification (Figure 3C). However, significantly more 27SB pre-rRNA was enriched with Pol5 than with the untagged or Utp10 controls (Figure 3C). In addition, the 35S and 32S pre-rRNAs were slightly enriched in the Pol5 purification when compared with the untagged control (Figure 3C and Supplementary Figure S4). These results indicate the association of Pol5 with both early pre-rRNAs and also with the LSU pre-rRNA 27SB.

### Pol5 is required for the recruitment of some RPs and AFs to pre-60S complexes

Our data indicate that Pol5 is required for the biogenesis of both ribosomal subunits, however, we first focused on further characterizing the role of Pol5 during pre-60S maturation. To explore a potential role for Pol5 in the recruitment of AFs and RPs during maturation of the 27S pre-rRNA, pre-ribosomal complexes were isolated via Noc2 or Rlp7 from cells either expressing or depleted of Pol5 for 7 h. Both Noc2 and Rlp7 bind early pre-60S particles, but Noc2 is released from 27SB containing particles before C2 cleavage whereas Rlp7 remains bound after processing at the C2 site (60,61). Thus, comparison of protein composition of Noc2 and Rlp7 particles allows changes in pre-ribosomal composition that occur before or after release of Noc2 to be identified. Affinity purification of Rlp7 revealed higher levels of Pol5 in the purified particles when compared with particles purified via Noc2 (Supplementary Figure S5A). These results indicate the earlier association of Noc2 with pre-ribosomal particles and a more robust association of Rlp7 with pre-ribosomal particles containing Pol5.

**Figure 4.**
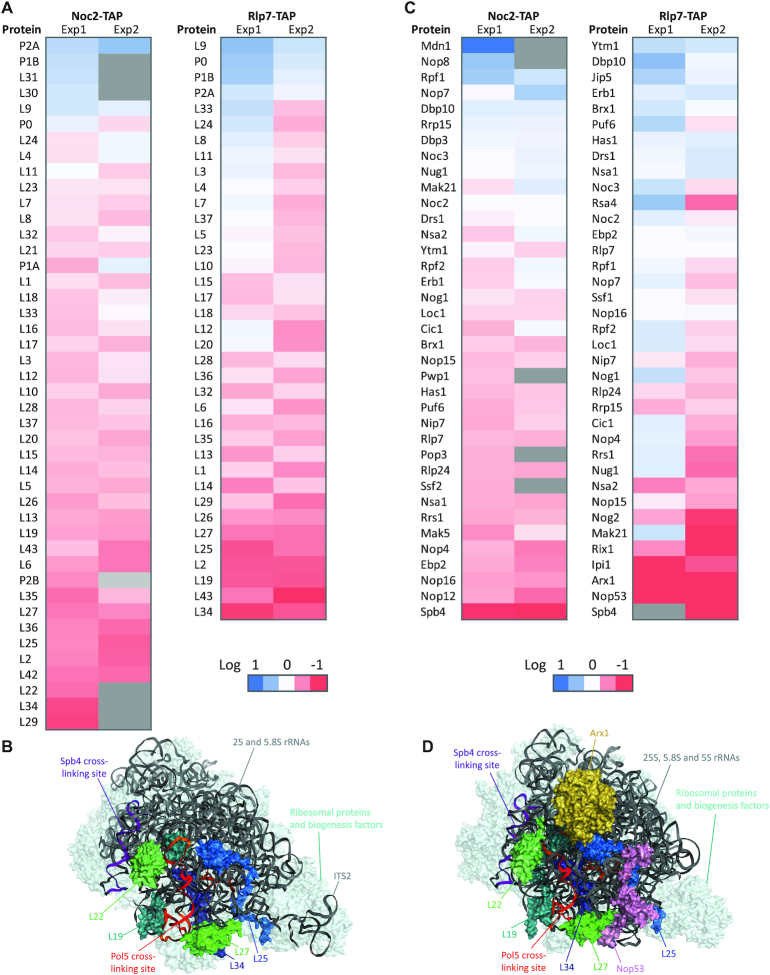
Depletion of Pol5 affects the recruitment of AFs and RPs that bind pre-60S complexes around the peptide exit tunnel. (**A**) Heatmap of LSU ribosomal proteins identified in the qMS analysis of Noc2- and Rlp7-associated particles. Only proteins identified in two independent experiments are shown (proteins not observed in the individual experiment are depicted in gray). iTRAQ ratios (Pol5 expression versus Pol5 depletion) were calculated to determine the relative abundance of proteins in the two samples. Color code indicates the log2 of iTRAQ ratios. (**B**) Structure of a pre-60S complex purified via Ytm1 (PDB: 6ELZ) with the binding site of Pol5 and RPs affected by Pol5 depletion highlighted. The rRNA is shown in cartoon view in dark gray and the crosslinking sites of Pol5 and Sbp4 are marked in red-yellow and purple respectively. RP and AFs are depicted in surface view, generally in pale cyan with the exception of ribosomal proteins depleted in pre-60S particles isolated from cells lacking Pol5, which are shown in colors and labeled. (**C**) Heatmap of AFs identified in the qMS analysis of Noc2- and Rlp7-associated particles. Color code as in A. (**D**) Structure of a pre-60S particle purified via Nog2 (PDB: 3JCT) with the binding sites of Pol5, Spb4 and selected AFs highlighted. Presentation styles and colors are as in (B) Domains IV and VI of 25S are indicated and separated by a dashed line.

In order to comprehensively and quantitatively analyze the associated proteome of Noc2 and Rlp7 in the presence and absence of Pol5, mass spectrometry analyses using iTRAQ^®^ were performed on the isolated particles (Supplementary Figure S5B). Depletion of Pol5 caused accumulation of components of the phosphostalk (P0, P1B, P2A; Figure 4A) in the Noc2- and Rlp7-containing particles. In contrast, upon Pol5 depletion, the Noc2 and Rlp7 associated particles contained decreased levels of a subset of RPs that associate with domain III of the 25S rRNA (L2 (uL2), L19, L22 (eL22), L25, L27, L34 (eL34) and L43 (eL43); Figure 4A). Interestingly, these RPs clamp helix h58 of the 25S rRNA sequence, which is bound by Pol5 (Figure 2C and D), within the body of pre-60S particles to form the outer face of the peptide exit tunnel (Figure 4B) (62). Assembly of domain III is a pre-requisite for site C2 cleavage (45,63) and therefore, the Pol5-dependent recruitment of RPs is likely an important milestone that enables ITS2 processing.

Furthermore, a number of AFs (Nop4, Nop12, Nop16 and Ebp2) were specifically depleted from the Noc2-associated particles in the absence of Pol5 (Figure 4C). However, depletion of these AFs was less significant in the Rlp7-associated particles. It is therefore tempting to speculate that Pol5 may contribute to the release of these factors from Noc2-containing particles. In contrast, Nop53 and Arx1 were strongly decreased in the Rlp7-associated particles (Figure 4C and D). Interestingly, the RNA helicase Spb4 was depleted from both the Noc2- and Rlp7-associated particles when Pol5 was lacking (Figure 4C). Although the pre-60S binding sites of Spb4 in ES27 (domain IV) and helix h101 (domain VI) of the 25S rRNA sequence (22,64,65) are spatially distinct from the Pol5 crosslinking site in domain III (Figure 4B and D), the available pre-60S structures show that maturation of domain III occurs prior to compaction of domains IV and VI of the 25S rRNA, suggesting that the binding of Pol5 to domain III may contribute to the structural reorganization of the 25S rRNA sequence, thereby facilitating formation of the appropriate interaction surface for Spb4. Depletion of Pol5 also caused strong decreases in the amounts of the late AFs Arx1 and Nop53 present in Rlp7-associated pre-60S particles. In the case of Arx1, the results are consistent with the requirement for Spb4 for recruitment of the pre-60S export factor Arx1 (64).

**Figure 5.**
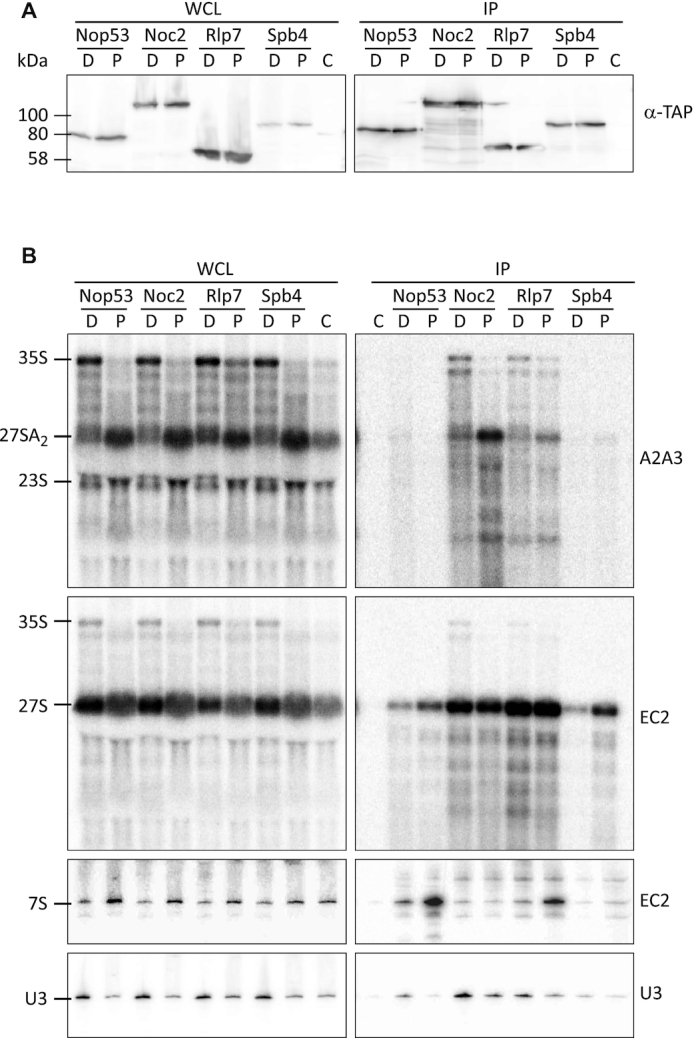
Depletion of Pol5 impairs the association of Spb4 and Nop53 with pre-rRNAs. (**A**) Pre-60S particles from different stages of 60S maturation were purified via TAP-tagged AFs either in the absence or presence of Pol5 (D and P respectively). Whole cell lysates (WCL) and affinity purification samples (IP) were analyzed by western blotting with the indicated antibodies. TAP-tagged proteins are indicated, C indicates the non-tagged strain BY4741 used as a control. (**B**) Northern blot analysis of pre-rRNAs co-purified with different AFs. RNAs enriched in the affinity purifications were extracted and resolved on denaturing agarose or acrylamide gels, transferred to nylon membranes, and hybridized with the indicated oligonucleotide probes.

To further characterize the impact of Pol5 on the recruitment of Spb4 and Nop53, complexes containing these AFs were affinity purified from cells expressing or lacking Pol5, alongside the particles associated with Noc2 and Rlp7 for comparison. Western blot analysis showed that depletion of Pol5 did not affect the levels of any of the bait AFs (Figure 5A) demonstrating that the stability of these AFs does not depend on Pol5. Consistent with our earlier findings (Figure 1), analysis of pre-rRNAs in the cell extracts (Figure 5B, left panels) by northern blotting showed decreases in the levels of the 27SA2 and 7S pre-rRNA species in cells depleted of Pol5 (Figure 5B, first and third panels from the top), as well as accumulation of the 35S and 27SB pre-rRNAs and the U3 snoRNA (Figure 5B, second and fourth panels from the top). As previously observed, affinity purified Noc2- and Rlp7-containing particles showed that Noc2 associates already with the 27SA2 pre-rRNA while Rlp7 predominantly enriches 27SB and remains associated with 7S-containing particles (Figure 5B, right panels). In line with the depletion phenotype of Pol5, which reduces the levels of the 27SA pre-rRNA species, Noc2 and Rlp7 were predominantly associated with the 27SB pre-rRNA upon depletion of Pol5. In addition, when Pol5 levels were reduced, the 35S and 33S pre-rRNA, as well as the U3 snoRNA, were enriched in the Noc2 and Rlp7 purifications, indicating the association of both AFs with 90S-like particles. In the case of Rlp7 and Nop53, Pol5 depletion led to decreased association of these AFs with the 7S pre-rRNA, which likely occurs due to inhibition of site C2 cleavage and therefore production of this pre-rRNA species in cells lacking Pol5. However, the mild enrichment of 27S pre-rRNAs with Nop53 suggests that this protein can still be recruited to pre-60S complexes when Pol5 is lacking, albeit at reduced levels. In contrast, affinity purification of Spb4 from cells expressing Pol5 showed the enrichment of the 27SB but not the 27SA2 pre-rRNA (48). However, as anticipated from the quantitative mass spectrometry analysis, after Pol5 depletion, the enrichment of 27SB with Spb4 purified particles was drastically reduced. As Spb4 dissociates from pre-60S subunits before C2 cleavage, this result could arise from Pol5-independent release of Spb4. However, this observation more likely reflects failure to recruit Spb4 to pre-60S complexes, due to impaired folding of domains IV and VI as previously mentioned. Altogether, these results suggest that Pol5 affects the recruitment of AFs Spb4, Arx1 and Nop53 but not of Noc2, Rlp7 and the Erb1 complex among other AFs.

### Pol5 is involved in the recycling of SSU processome components

**Figure 6.**
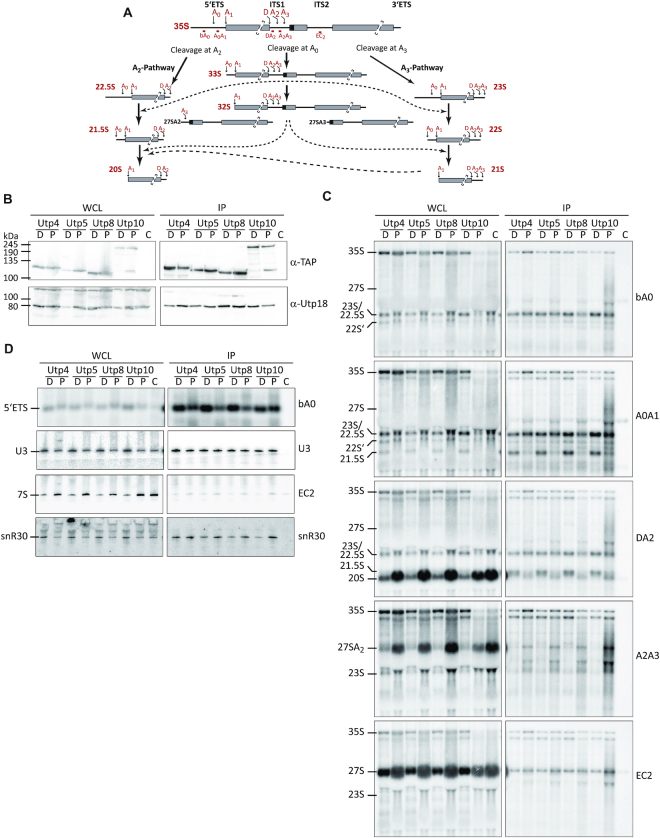
Depletion of Pol5 affects the release of t-Utp proteins from 5′ ETS-containing pre-rRNAs. (**A**) Schematic representation of alternative pre-rRNA processing pathways for removal of the 5′ ETS sequences of the 35S pre-rRNA. Positions of the primers used for northern blot analysis are indicated and named by the region where they hybridized. (**B**) Complexes associated with TAP-tagged t-Utps were purified in the absence or presence of Pol5 (D and P respectively). Whole cell lysates (WCL) and affinity purification samples (IP) were analyzed by western blot with indicated antibodies. TAP-tagged proteins are indicated, C indicates the non-tagged strain BY4741 used as a control. (**C**, **D**) Northern blot analysis of RNAs co-purified with t-Utps. RNAs enriched in the affinity purifications were extracted and resolved on denaturing agarose (C) or acrylamide (D) gels, transferred to nylon membrane, and hybridized with the indicated oligonucleotide probes.

Pol5 was initially identified with other t-UTP components as part of the UTP-A complex (38) and here, we reveal Pol5 contacts with the 5′ ETS that clustered at the binding site of the t-UTP complex (Figure 2E). The 5′ ETS is involved in nucleation of AFs of the SSU processome (66–68), a process that is initiated by association of the t-Utps (5,6,11). Pre-rRNA processing at sites A0, A1 and A2 occur within the SSU processome, and perturbation of any of these cleavage events can lead to the use of alternative pre-rRNA processing pathways and the accumulation of aberrant pre-rRNA species (Figure 6A). The association of Pol5 with the 5′ ETS, together with the finding that Pol5 is required for cleavages at the A0, A1 and A2 sites (Figure 1C), suggests a function of Pol5 in the context of the SSU processome. Potential roles of Pol5 in the recruitment of SSU AFs and in the processing or turnover of SSU pre-rRNA were therefore investigated.

The t-Utp proteins, Utp4, Utp5, Utp8 and Utp10 were TAP-tagged in the yeast strain that conditionally expressed Pol5. Western blot analysis of cell extracts upon expression of exogenous Pol5 or depletion showed similar levels of all TAP-tagged t-UTP proteins, confirming that their stability is not affected by lack of Pol5 (Figure 6B). Moreover, depletion of Pol5 did not impair the association of any of the t-Utps and the UTP-B component, Utp18, suggesting that formation of the SSU processome is not impaired by lack of Pol5.

Northern blot analysis of RNAs from cell extracts again confirmed the decrease in levels of 27SA2 and 7S pre-rRNAs as well as the accumulation of the 35S and 27SB pre-rRNAs upon depletion of Pol5. In addition, accumulation of 22.5S and 21.5S pre-rRNAs, and a concomitant reduction of the 20S pre-rRNA was observed upon Pol5 depletion (Figure 6C, left panels, compare DA2 with A2A3 panels). This result is consistent with the necessity for Pol5 for efficient A0, A1 and A2 cleavages and with the strong decrease of 20S observed in the pulse-chase experiment (Supplementary Figure S1E). Surprisingly, we detected the presence of aberrant pre-rRNAs (22S’) with bA0 probe but not DA2 or A2A3 (Figure 6C). Based on their size, these pre-rRNAs could be produced by cleavage at an unknown processing site upstream the A2 site or at site D, which would indicate the anomalous transport of non-processed pre-rRNAs to the cytoplasm. Analysis of pre-rRNAs associated with Utp4, Utp5, Utp8 and Utp10 showed that 35S and 22.5S pre-rRNAs were enriched with the t-Utp proteins independently of Pol5 (Figure 6C). Interestingly, the 33S and 21.5S pre-rRNAs accumulated in the t-Utp purifications when Pol5 was depleted (Figure 6C). This result is striking as both these pre-rRNA species lack the 5′ end of the 35S transcript and therefore, the binding site for the t-Utp proteins. Moreover, the 5′ ETS fragment generated by cleavage at A0 was also enriched with the t-Utp proteins upon Pol5 depletion (Figure 6D, upper panel). These results suggest that when Pol5 is lacking, the initial pre-rRNA transcript can undergo cleavage at sites A0 and A2, but that the pre-rRNA fragments generated remain associated with each other via protein-protein interactions. Failure to release these pre-rRNA fragments from the t-UTP proteins will impair recycling of the t-Utps thereby inhibiting their recruitment to nascent transcripts, likely causing the defects in synthesis of SSU observed upon depletion of Pol5. Notably, although Pol5 crosslinks to the 5′ ETS (Figure 2C and E), the 5′ ETS fragment generated by A0 cleavage was not enriched with Pol5 implying that Pol5 associates with this region in the context of the 35S pre-RNA. While we cannot exclude a weak or transient interaction of Pol5 with this fragment, our data rather suggest an indirect role of Pol5 in promoting the recycling of t-UTP proteins from excised pre-rRNA fragments.

## DISCUSSION

### Association of Pol5 with pre-rRNAs and role in processing

Although Pol5 and its orthologs have been suggested to participate in ribosome biogenesis (34,36), their precise roles in the synthesis of ribosomes have been little characterized so far. Our affinity purifications suggested the association of Pol5 with the 35S and 27SB pre-rRNAs, and using CRAC, we have uncovered contact sites between Pol5 and these pre-rRNAs. Our data indicated that Pol5 contacts the ITS2 region and helix h58 within the domain III of the 25S sequence, which is in line with the requirement for Pol5 for LSU biogenesis. Notably, Pol5 contacts ITS2 near the C2 cleavage site, and although the ITS2 sequences bound by Pol5 are not visualized in the available cryo-EM structures of pre-60S particles (41,62,65), structure probing data indicates a preferential orientation of the central domain of ITS2 toward L27 and domain III (69). Our data also indicated contacts between Pol5 and the 5′ ETS of the 35S pre-rRNA, which likely correlate with the original description of Pol5 as component of the UTP-A complex (38). Depletion of Pol5 leads to accumulation of the 35S pre-rRNA, probably due to impaired cleavage at site A2 in ITS1. A mild decrease in the level of the 27SA3 pre-rRNA was also observed upon depletion of Pol5, however, as the 23S pre-rRNA that is also generated by A3 cleavage accumulated, these data suggest that while A3 processing may be less efficient in the absence of Pol5, it is not inhibited. The difference in the effect of Pol5 depletion on the 27SA3 and 23S pre-rRNAs can be explained by the differences in the stability of these intermediates; while 27SA3 is rapidly processed to 27SB by the Rat1 exonuclease (70), titration of the tUtps caused by the absence of Pol5 may block further processing of the 23S pre-RNA which accumulates readily until it can be degraded by the surveillance machinery (10,55). In line with this, when Pol5 was depleted, a slight accumulation of the 27SB pre-rRNA was observed. Interestingly, Pol5 associates preferentially with 27SB pre-rRNAs and, upon depletion of Pol5, we did not observe alterations in the levels of any of the A3 AFs on pre-60S particles nor changes in the ratio of the 27SB_S_/B_L_ expected from the absence of an A3 AF(70).

### Pol5 is involved in formation of the polypeptide exit tunnel

By performing qMS, we observed reduced levels of several RPs (L2, L19, L22, L25, L27 and L34) on purified pre-60S particles in the absence of Pol5. These RPs cluster around the binding site of Pol5 in helix h58 within the domain III of the 25S rRNA (Figure 2 and 3) and are components of the polypeptide exit tunnel (PET) (41,62,65). Interestingly, during the review process of this manuscript, the *L25* and *L27A* genes have been described as multicopy suppressors of temperature sensitive mutants of Pol5 (37), further supporting the association of Pol5 with this pre-rRNA region. As the incorporation of RPs that participate in formation of the PET precedes the cleavage of the ITS2 (45), it is likely that failure to recruit these proteins causes the defect in C2 cleavage observed upon depletion of Pol5.

Considering the different states observed in the available cryo-EM structures of pre-60S particles as different assembly intermediates (41,65), recruitment of L25 appears to occur at the transition between states 2 and 3 of the Nsa1-Nop2 particles (65). Moreover, the stable association of L25 would require compaction of domain III that takes place in the transition from state C to state D in the Ytm1 purified particles (41). Our results suggest that assembly of at least L25 would require the action of Pol5. It is possible that the association of Pol5 with domain III traps L25 between domains I and III. Interestingly, depletion of L25 has been described to cause the specific depletion of other RPs that form the PET (L2, L19, L31, L34 and L43) (39). Thus, it may be that reduced levels of these RPs upon depletion of Pol5 is a secondary effect caused by lack of L25.

Strikingly, neither depletion of Pol5 nor lack of L25 affects recruitment of AFs found in the periphery of the N-terminal domain of L25 (Erb1, Nop7 and Rlp7) (39,41,62). Moreover, recruitment of L27 is reduced in the absence of Pol5 and it could be independent of L25 (39). As observed in the structure of a pre-60S particle purified via Nog2 (62), L27 and Nop7 constitute an interaction surface for Nop53. In earlier pre-60S particles, L27 and Nop7, together with the helix 58 of the 25S rRNA, establish contacts with Erb1 instead of Nop53 (states D and E of the Ytm1 particle; 62). It is possible that L27, together with L25, participates in compaction of domain III during the transition from state C to state D (41), required for the exact positioning of Erb1 during LSU formation. Continuing rearrangement of domain III may then assembly helix h58 in its mature conformation, possibly facilitating dissociation of Erb1 by the action of the AAA-ATPase Rea1 (57,71). Therefore, the positioning of L25 and L27 may trigger a cascade of interactions with other RPs that bind around the PET, which promotes assembly into the mature structure.

Among the AFs depleted from pre-ribosomal particles in cells lacking Pol5, was the RNA helicase Spb4. It is possible that Spb4 is released from the pre-ribosomal particles accumulated upon depletion of Pol5. However, this is unlikely because the binding site of Spb4 is formed by domains IV and VI of the 25S rRNA sequence (64). Moreover, the protein L2, which is present at lower levels on pre-60S complexes when Pol5 is depleted, might be required for the correct position of domain IV according to the structures of pre-ribosomal particles (41,62). In agreement with this model, the binding site of Spb4 starts to be observed in state E of the Ytm1-associated particles (41). Therefore, state E, which already contains L25 but not L2, is likely the earliest pre-60S complex with which Spb4 can associate. We also observed a clear reduction of Nop53 in particles purified via Rlp7 in the absence of Pol5. However, our results indicated a weak association of Nop53 with 35S and 27SA pre-rRNAs. It is possible that seclusion of Erb1 in the 27SB particles allows premature recruitment of Nop53 by protein–protein interactions (possibly via Nop7). However, the presence of Erb1, the lack of L27, and defects in the assembly of domain III might not support the stabilization of Nop53. Interestingly, not only Spb4 but also L25 and L19 are required for recruitment of the AF Arx1 at the base of helix 59 (62). Consistent with this, our data showed a clear decrease in the recovery of Arx1 in the Rlp7 purified particles upon depletion of Pol5. Accordingly, formation of the PET is required for the export of pre-LSU particles (22). Another striking result was the increase in pre-ribosomal particles containing the phosphostalk proteins upon depletion of Pol5, as PET maturation and phosphostalk assembly seem to be connected (72). However, the link between the assembly of both these structures relies on the release of Mtr4 a protein involved in, but not essential for, the assembly of the phosphostalk (73,74).

### Pol5 affects the disassembly of the SSU processome

Interestingly, when Pol5 was depleted, most of the RPs of the SSU were up to 2-fold enriched in the particles isolated via Noc2 and Rlp7 when compared with cells expressing Pol5 (Supplementary Figure S5C). Accordingly, enrichment of the 35S pre-rRNA with these AFs also increased upon depletion of Pol5. Defects in LSU assembly have been described to have feedback effects on SSU assembly (58,75,76). This may be explained by the fact that early ribosome assembly steps occur within the context of a large 90S pre-ribosomes containing both SSU and LSU biogenesis factors (76), however, the mechanism(s) underlying the inter-dependency of subunit assembly are not yet fully understood.

The association of Pol5 with t-Utp proteins and the contact sites of Pol5 in the 5′ ETS, together with the role of Pol5 in PET formation in the LSU, suggests a potential role for Pol5 in connecting the assembly of both ribosomal subunits. The accumulation of 35S and 23S pre-rRNAs when Pol5 was lacking indicate that pre-rRNA processing occurs by post-transcriptional cleavage at the A3 site to produce the 23S pre-rRNA (77). As Pol5 depletion does not seem to cause a drastic reduction in 40S levels, it is likely that the defects in pre-40S assembly observed when Pol5 is lacking are indirect and our data suggest that they may arise due to inefficient recycling of t-Utp proteins from excise 5′ ETS pre-rRNA fragments. Remarkably, the t-Utp proteins are the only SSU-processome components that are also required for LSU assembly (5,6). Although this may be due to their putative function during transcription of the rDNA, it is also possible that this dual function reflects their participation in the recruitment of LSU AFs, such as Pol5. A recent work published during the preparation of this manuscript suggests that association of Pol5 with the t-Utp proteins to form the UTP-A complex occurs prior to formation of the SSU processome (78).

Interestingly, although more than 200 AFs are involved in ribosome assembly in yeast, only few are required for the maturation of both subunits. Among them, are the helicases Has1 and Prp43. Has1 is required for release of the U14 snoRNP from the early pre-SSU complexes and promotes assembly of domain I of the LSU (79–81), whereas Prp43 is implicated in release of a number of snoRNPs from early pre-LSU complexes as well as facilitating access of the endonuclease Nob1 to its cleavage site at the 3′ end of the 18S rRNA during the late stages of pre-SSU maturation (82,83). For both proteins, the positions and timings of these dual functions suggest that they likely represent independent binding events and functions. In contrast, the large protein Rrp5 acts as a scaffold within early pre-ribosomal complexes, simultaneously forming rRNA contacts that are important for the maturation of both subunits (84–86). Although Pol5 similarly binds to rRNA sequences linked to SSU (5′ ETS) and LSU (ITS2 and 25S rRNA sequence) biogenesis, it currently remains unknown whether they reflect different contact sites of a single Pol5 molecule or alternatively, separate binding events. Either way, coordinating dissociation of the t-UTP proteins from 5′ ETS fragments within the SSU processome with maturation of 60S subunits may contribute to regulating the stoichiometric production of ribosomal subunits.

## DATA AVAILABILITY

GEO database: GSE132973.

## Supplementary Material

gkz1079_Supplemental_File
