# Supplementary material for: Pol5 is required for recycling of small subunit biogenesis factors and for formation of the peptide exit tunnel of the large ribosomal subunit
Source: Nucleic Acids Res. 2019 Nov 20;48(1):405–20. doi: 10.1093/nar/gkz1079 (PMC7145529; doi:10.1093/nar/gkz1079)
Supplement: gkz1079_Supplemental_File [file gkz1079_supplemental_file.pdf]

Pol5 is required for recycling of small subunit biogenesis factors and for formation of the peptide exit tunnel of the large ribosomal subunit

Christina M. Braun, Philipp Hackert, Catharina E. Schmid, Markus T. Bohnsack, Katherine E. Bohnsack, and Jorge Perez-Fernandez

Supplementary figures

Supplementary Figure S1. Depletion of Pol5 impairs 60S biogenesis.

(A) Wild type yeast cells (BY) or cells from the *GAL::HA-POL5* strain (GAL) were grown in exponential phase in SCG media before harvesting. RNAs were isolated, separated by denaturing agarose and acrylamide gels then transferred to nylon membranes. Northern blotting was performed using [^{32}P]-labelled probes as indicated. **(B)** Wild type yeast (WT) and cells from the *GAL-HA-POL5* strain transformed with a plasmid for exogenous expression of *POL5* were cultivated in SCG or SCD for 24 h and the doubling times of were determined by monitoring the optical density at 612 nm. **(C)** Growth curve of strains in which genomically encoded *POL5* is under the control of a galactose-inducible/glucose-repressible promoter. Cells containing a plasmid encoding *POL5* (Pol5) or an empty plasmid backbone (Depletion) were cultivated in YPD for 16 h. Cells were kept in exponential growth phase by diluting the cultures every 6 h. **(D)** 5 AU of cells from the *GAL::HA-POL5* strain were collected at the indicated times (h) of culturing in glucose, proteins were extracted and analyzed by western blotting. Depletion of Pol5 was analyzed using an anti-HA antibody and western blotting using an anti-tubulin antibody served as a loading control. **(E)** Exponentially growing cells from the *GAL::HA-POL5* strain carrying either a plasmid for expression of *POL5* (Pol5) or the empty plasmid backbone (Depletion) were grown in SCD media for either 3 or 7 h. Cells were then metabolically labelled with [5,6-^{3}H]-uracil for 5 min and then chased with an excess of unlabelled uracil. Total RNA was extracted and separated by denaturing agarose (upper panel) and acrylamide (lower panel) gel electrophoresis. The (pre-)rRNA species corresponding to each of the bands detected are indicated. **(F, G)** Sucrose gradient analysis of the effect of Pol5 depletion on ribosomal subunit and polysome production. Cells containing a plasmid encoding *POL5* (Pol5) or an empty plasmid backbone (Depletion) were cultured for 9 h in SCD to deplete endogenous Pol5. Cells were incubated or not with cycloheximide and soluble lysates were prepared. Cell lysates obtained in the absence of cycloheximide were resolved in low magnesium sucrose gradients (10 to 40%) to analyze the content of ribosomal subunits (F). Cell lysates prepared from cells treated with cycloheximide were resolved in sucrose gradients (10 to 40%) to analyse their polysome content (G). Profiles were obtained by recording absorbance at 260 nm during fractionation.

Supplementary Figure S2. Depletion of Pol5 impairs synthesis of 25S and 7S pre-rRNAs.
(A) Northern blot analysis of pre-RNAs. Total RNAs obtained at indicated depletion times (hours) were resolved in denaturing agarose (top panel) or acrylamide (bottom panels) gels, transferred to nylon membranes, and hybridized with the indicated oligonucleotide probes. **(B)** Primer extension analysis of pre-RNAs to detect the 25.5S/26S pre-rRNA species during Pol5 depletion. Total RNAs obtained at the indicated depletion times (hours) were used in a primer extension assay with the C2C1 oligonucleotide. Reaction products were separated by denaturing polyacrylamide gel electrophoresis and radioactively labelled cDNA fragments were detected. Sequencing reactions on a plasmid (K375) encoding a full ribosomal DNA copy were performed in parallel (lanes G, A, T, and C). A longer exposure of the gel area indicated by a dotted line is presented to enable visualization of signals derived from the 25.5S/26S pre-rRNA intermediate generated by C2 cleavage.

Supplementary Figure S3. Mapping of the Pol5 crosslinking sites on a pre-60S complex.
The number of sequencing reads mapping to each nucleotide of the 25S rRNA in the Pol5-HTP CRAC dataset is shown on the tertiary structure of the pre-60S rRNAs (PDB 6ELZ; **(1)**) using a color scale in which the maximum number of reads (100%) is shown in red and lower numbers of reads are shown in yellow (30%).

Supplementary Figure S4. Association of Pol5 and other AFs with pre-rRNAs.
Ip samples from Pol5 and Control (C) probed with bA0, A0A1 and A2A3. Northern blots shown in Figure 3C are shown in a longer exposure.

Supplementary Figure S5. Depletion of Pol5 causes accumulation of RPs and AFs of the SSU in Noc2- and Rlp7-containing particles.
(A) Complexes associated with TAP-tagged Noc2 and Rlp7 were purified either in the absence or presence of Pol5 (D and P respectively). Whole cell lysates (WCL) and affinity purification samples (IP) were analysed by western blot with indicated antibodies. C indicates the non-tagged strain BY4741 used as a control. **(B)** Proteins co-affinity purified TAP-tagged Noc2 or Rlp7 from cells expressing (P) or depleted (D) of Pol5 were resolved on a denaturing acrylamide gradient gel (4-12%) and stained with SimplyBlue™ SafeStain. **(C)** Heatmap of ribosomal proteins and AFs of the small subunit identified in the qMS analysis of Noc2- and Rlp7-associated particles. Only proteins identified by at least two peptides in two independent experiments are shown. The iTRAQ ratio (Pol5 expression *vs* Pol5 depletion) was calculated to determine the relative abundance of proteins

in the two conditions. Data were normalized by the ratio of the bait proteins. Color code indicates the log2 of iTRAQ ratios. Proteins not observed in the individual experiment are depicted in grey.

Supplementary tables

Table S1: Oligonucleotides used in this study. Oligonucleotides marked with "o" belongs to the collection of the UTP-Lab. Oligonucleotides marked with "#" belong to the department of Biochemistry III at the University of Regensburg. Oligonucleotide marked oMB belong to the Bohnsack lab, UMG.

Col. N.	Name	5'-3'-sequence	Purpose
**o67**	vA0	CGCTGCTCACCAATGG	NB probe, hybridizes in the rRNA, 5'ETS
**o86**	FLAG_POL5_F	ATGGATTACAAGGATGACGACGATAAGGGTACCGGATCCATGACAGGGAAAGTCAACAGAG	forward primer used to amplify *POL5* from genomic DNA of BY4741 for cloning into pCM182-LEU2
**o87**	NotI_POL5_Re	GGAAACAGCTATGACCATGATTACGCCAAGCTTGCATGCGCGGCCGCAGGTATGGACTCGTATGTTTATC	reverse primer used to amplify *POL5* from genomic DNA of BY4741 for cloning into pCM182-LEU2
**o127**	Spb4_S3_fw	CGGAAGAAAGTTTCCAGCAAAGCTATCCAAGGCAATTTTGACGACTTACGTACGCTGCAGGTCGAC	forward primer used to amplify TAP tag from pYM15-TAP-URA for yeast homologous recombination at the C-terminus of *SPB4*
**o128**	Spb4_S2_rev	CCATTGGTTAAGAATGTTGAGTGATTCTACGAACAAGGTAACTTTTTTTCCATAAATCGATGAATTCGAGCTCG	reverse primer used to amplify TAP tag from pYM15-TAP-URA for yeast homologous recombination at the C-terminus of *SPB4*
**#205**	o2-18S	CATGGCTTAATCTTTGAGAC	NB probe, hybridizes in the rRNA, 18S
**#207**	o4-A2/A3	TGTTACCTCTGGGCCC	NB probe, hybridizes in the rRNA, ITS1
**#208**	o5-A3/B1	AATTTCCAGTTACGAAAATTCTTG	NB probe, hybridizes in the rRNA, ITS1
**#210**	o7-E/C2	GGCCAGCAATTTCAAGTTA	NB probe, hybridizes in the rDNA, ITS2
**#211**	o8-C1/C2	GAACATTGTTCGCCTAGA	NB probe, hybridizes in the rDNA, ITS2
**#212**	o9-25S	CTCCGCTTATTGATATGC	NB probe, hybridizes in the rDNA, 25S
**#621**	Noc2-TAP FP	AAGTGATGATGACAACGAAGATGTTGAAATGTCAGACGCTTCCATGGAAAAGAGAAG	forward primer used to amplify TAP tag from pBS1539 for yeast homologous recombination at the C-terminus of *NOC2*
**#622**	Noc2-TAP RP	CTATTGAATTCAAGACAAAAAATCAAATCTTGCTGAGTTGTACGACTCACTATAGGG	reverse primer used to amplify TAP tag from pBS1539 for yeast homologous recombination at the C-terminus of *NOC2*
**#1813**	Utp4-TAP-F	ACTTTTCACTCCAAACAAAAGGCGTTTATTCAACCAAAGTTAGTGTTTTCCATGGAAAAGAGAAG	Forward primer for amplification of TAP-tag from pBS1539
**#1814**	Utp4-TAP-R	GCCTTTTAATAGCATCTCTCTATTCTTCGGTATGTTGACTTAAATTAATACGACTCACTATAGGG	Reverse primer for amplification of TAP-tag from pBS1539
**#2474**	5S-rDNA	CAGCGGGTACTCCTACCTGATT	NB probe, hybridizes in the rDNA, 5S
**#2921**	A0A1	CCCACCTATTCCCTCTTGCTAG	NB probe, hybridizes in the rDNA, 5'ETS
**#2934**	NOP53-TAP_fw	AGTGCCCGTTAGGAAAGGTAGAAAGTATAAGCAGAAAATCACTGAAAAGTGGACACATAAGGACTTCAAATCCATGGAAAAGAGAAG	forward primer used to amplify TAP tag from pBS1539 for yeast homologous recombination at the C-terminus of *NOP53*

**#2935**	NOP53-TAP_rev	ATCTCACTTGATGAATCCACGTATCAAGGACAACCTTTTCATGGAAACATATACTGTAAAACAAAAAACTTACGACTCACTATAGGG	reverse primer used to amplify TAP tag from pBS1539 for yeast homologous recombination at the C-terminus of *NOP53*
**#2959**	5.8S rRNA 3'probe	AAATGACGCTCAAACAGGCAT	NB probe, hybridizes in the rDNA, 5.8S
**#3468**	SnR17-Middle	CCGCTAAGGATTGCGGACCAAGC	NB probe, hybridizes in the U3 snoRNA
**#3469**	SnR30-Middle	CAGAGGAGAAGTCAGGAGCTGC	NB probe, hybridizes in the snR30 snoRNA
**#3696**	UTP5-S3-fw	AGCGACGGCGAGGAGGAAGCCGGATATAGTGACGTTGAGATGGAACGTACGCTGCAGGTCGAC	forward primer used to amplify TAP tag from pYM15-TAP-URA for yeast homologous recombination at the C-terminus of *UTP5*
**#3697**	UTP5-S2-rev	ATTTTTGTATTCTGATGCGTGAAAGCATTTTATGCATGATATCCTATCGATGAATTCGAGCTCG	reverse primer used to amplify TAP tag from pYM15-TAP-URA for yeast homologous recombination at the C-terminus of *UTP5*
**#3698**	UTP8-S3-fw	CAGAAGCGAGCCTTACCCACTTACACCATGGAATACTTGGACATTCGTACGCTGCAGGTCGAC	forward primer used to amplify TAP tag from pYM15-TAP-URA for yeast homologous recombination at the C-terminus of *UTP8*
**#3699**	UTP8-S2-rev	TTTTCTATATAGGTATTATACAATACAATCAAATTCATTGCATACATCGATGAATTCGAGCTCG	reverse primer used to amplify TAP tag from pYM15-TAP-URA for yeast homologous recombination at the C-terminus of *UTP8*
**#3702**	UTP10-S3-fw	GTTGTTGAAAACGTTTTAGGGGAACCTTTTGATAGGTATTTAGATCGTACGCTGCAGGTCGAC	forward primer used to amplify TAP tag from pYM15-TAP-URA for yeast homologous recombination at the C-terminus of *UTP10*
**#3703**	UTP10-S2-rev	GTGTTTTACTTTACAAAAATTTACATAATACTTCACCTTTTTTTTATCGATGAATTCGAGCTCG	reverse primer used to amplify TAP tag from pYM15-TAP-URA for yeast homologous recombination at the C-terminus of *UTP10*
**#3839**	ITS1 D-A2 mid	AAGCCTAGCAAGACCGCGCA	NB probe, hybridizes in the rDNA, ITS1
**#3883**	S3_TAP	CAGCTGAAGCTTCGTACGCTGCAGGTCGACATGGAAAAGAGAAGATGGAAAAAG	Forward primer for amplification of TAP::URA3 from pBS1539
**#3884**	S2_TAP	GGATCTGATATCATCGATGAATTCGAGCTCGATACGACTCACTATAGGGCG	Reverse primer for amplification of TAP::URA3 from pBS1539
**#4012**	POL5_F4	GGGTGATTCTTTCCACAAGTGCATCTTTTTGTCCACAAAAAAAGAATTCGAGCTCGTTTAAAC	forward primer used to amplify the KANMX::GAL::HA cassette from pFA6a-kanMX6-PGAL1-3HA for insertion in the *POL5 locus*.
**#4013**	POL5_R3	CAGAAGCTAGTTTAAAAAAAAGGTCTCTGTTGACTTTCCCTGTCATGCA CTGAGCAGCGTAATCTG	reverse primer used to amplify the KANMX::GAL::HA cassette from pFA6a-kanMX6-PGAL1-3HA for insertion in the *POL5 locus*.
**#4072**	RLP7_For_Int	CCAGTTATCGAAGTTGACATTGACTCTTTATTAGCCAAGTTGAATTCCATGGAAAAGAGAAG	forward primer used to amplify TAP tag from pBS1539 for yeast homologous recombination at the C-terminus of *RLP7*
**#4073**	RLP7_Rev_Int	TAACTAACAACCTATGTACTATACAATTTTAAAATACTCTCTTAATACGACTCACTATAGGG	reverse primer used to amplify TAP tag from pBS1539 for yeast homologous recombination at the C-terminus of *RLP7*
**oMB6909**	Pol5-cHTP_fw	CATTTTTATTGATTTCATCAATTGGCTATCTTCAAAAAAGCAA	forward primer used to amplify HTP tag from pBS1539 for yeast

		ACTGTAATGGATAAGGAATCC ATGGAGCACCATC	homologous recombination at the C-terminus of *POL5*
**oMB6910**	Pol5-cHTP_rev	CGTAATTAAACAAAGATCAAC ATACATAATCCGATTTTGAGG AGTGATAAAATATACTTAGAT ACGACTCACTATAGGGCGA	reverse primer used to amplify HTP tag from pBS1539 for yeast homologous recombination at the C-terminus of *RLP7*

Table S2: Plasmids used in this study. Plasmids marked with "p" belongs to the collection of the UTP-Lab. Plasmids marked with "K" belong to the department of Biochemistry III at the University of Regensburg. The plasmid marked "pMB" belongs to the Bohnsack lab, UMG.

Col. N.	Name	Gene	Marker	Cloning procedure
**K1**	pBS	empty backbone plasmid	Amp	
**K97**	pBS1539	TAP::URA3	Amp/URA3	(2)
**K375**	pT11	rDNA	Amp	
**K1224**	pYM15	TAP::KANMX	Amp	(3)
**K1837**	pCM182-LEU2	empty backbone plasmid	Amp/LEU2	LEU2 marker replaces TRP1 in pCM182 (4, 5)
**p96**	pYM15-TAP-URA	TAP::URA3 with S2-S3 primer	Amp	TAP cassette from pBS1539 was inserted in pYM15 using oligo-nucleotides #3883 and #3884
**p181**	pFA6a-kanMX6-PGAL1::3HA	KANMX6-PGAL1::3HA	Amp	(6)
**p199**	ptCMS1	*POL5*, tetOFF	Amp/LEU2	PCR product obtained with oligonucleotides o86-o87 ligated into pCM182-LEU2 digested with BamHI-NotI
**p202**	ptDLS5	FLAG-*RPA12DE*, tetOFF	Amp/LEU2	pCM182-LEU2 plasmid containing FLAG epitope
**p203**	ptCMS2	FLAG-*POL5*, tetOFF	Amp/LEU2	[BamHI-NotI]-fragment from ptCMS1 subcloned in ptDLS5
**pMB439**	pBS1539	HTP::HIS3	Amp/HIS3	

Table S3: Yeast strains used in this study. Yeast strains marked with "Y" belongs to the collection of the UTP-Lab. Yeast strains marked with "#" belong to the department of Biochemistry III at the University of Regensburg. The yeast strain marked with "yMB" belongs to the Bohnsack lab, UMG.

Col. N.	Name	Genotype	Origin
**#206**	BY4741	MATa; his3-1; leu2-0; met15-0; ura3-0	Biochemie III
**#1879**	BY4742 NOC2-TAP	MATa; his3-1; leu2-0; ura3-0; lys2-0 YOR206W-TAP (URA3-KL)	Biochemie III
**#3197**	YJPF195-1a	MATa; his3-1; leu2-0; met15-0; ura3-0; POL5-TAP::URA3	This study
**Y281**	YMH5-1a	MATa; his3-1; leu2-0; met15-0; ura3-0; KANMX::GAL::HA-POL5	This study
**Y283**	YMH6-1a	MATa; his3-1; leu2-0; met15-0; ura3-0; KANMX::GAL::HA-POL5; UTP4-TAP::URA3	This study
**Y357**	YCMS7-1a	MATa; his3-1; leu2-0; met15-0; ura3-0; KANMX::GAL::HA-POL5; UTP5-TAP::URA3	This study

**Y372**	YCMS15-1a	MATa; his3-1; leu2-0; met15-0; ura3-0; KANMX::GAL::HA-POL5; NOP53-TAP::URA3	This study
**Y373**	YCMS16-1a	MATa; his3-1; leu2-0; met15-0; ura3-0; KANMX::GAL::HA-POL5; NOC2-TAP::URA3	This study
**Y377**	YCMS20-1a	MATa; his3-1; leu2-0; met15-0; ura3-0; KANMX::GAL::HA-POL5; UTP8-TAP::URA3	This study
**Y379**	YCMS22-1a	MATa; his3-1; leu2-0; met15-0; ura3-0; UTP10-TAP::URA3	This study
**Y380**	YCMS23-1a	MATa; his3-1; leu2-0; met15-0; ura3-0; KANMX::GAL::HA-POL5; UTP10-TAP::URA3	This study
**Y383**	YCMS24-1a	MATa; his3-1; leu2-0; met15-0; ura3-0; RLP7-TAP::URA3	This study
**Y384**	YCMS25-1a	MATa; his3-1; leu2-0; met15-0; ura3-0; KANMX::GAL::HA-POL5; RLP7-TAP::URA3	This study
**Y390**	YCMS27-1a	MATa; his3-1; leu2-0; met15-0; ura3-0; KANMX::GAL::HA-POL5; SPB4-TAP::URA3	This study
**yMB1599**	BY4741 POL5-HTP	MATa; his3-1; leu2-0; met15-0; ura3-0; POL5-HTP::HIS3	This study

Table S4: Antibodies used in this study.

Antibody	Origin	Dilution	Origin
**Anti-FLAG**	rat	1:1000	Agilent Tech. (200473)
**Anti-HA**	rat	1:2000	Roche (3F10)
**Anti-PAP (peroxidase conjugated)**	rabbit	1:5000	GenScript (A01435-100)
**Anti-Utp18**	rabbit	1:1000	Agro-Bio
**Anti-Tubulin**	rat	1:5000	Abcam (ab6161)
**Anti-rabbit (peroxidase conjugated)**	goat	1:5000	Dianova (111-035-003)
**Anti-rat (peroxidase conjugated)**	goat	1:5000	Dianova (112-035-068)

Supplementary methods

Growth curve.

Yeast strain YMH5-1a (Y281) was transformed with either a plasmid backbone (pCM182-*LEU2*; depletion) or the equivalent plasmid for expression of *FLAG-POL5* (ptCMS2; WT). Both strains were cultivated in galactose-containing minimal medium lacking leucine (SCG-Leu) and exponentially growing cells were inoculated in SCD-Leu. For each strain, the absorption units (AU) at 600 nm were measured after 0, 1.5, 3, 6, 9, and 16 h. Cultures were maintained in exponential growth phase by dilution in fresh medium every 6 h. 20 AUs of cell cultures were collected each time for protein and RNA analysis.

The growth rate of wild type yeast (BY4741) and the YMH5-1a (*GAL-HA-POL5)* strain was monitored by measuring the increase in optical density upon cultivation in 96-well plates for 24 h at 30°C in medium containing either glucose or galactose using a Tecan (Infinite 500) reader.

TCA protein precipitation method.

Proteins were precipitated from whole cell lysates prepared from cells harvested during Pol5 depletion experiments as previously described (7).

Pulse-chase analysis of nascent RNAs with [^{3}H]-Uracil.

Metabolic labelling of the YMH6 strain expressing an extrachromosomal copy of *POL5* from the ptCMS2 plasmid or containing an empty vector (pCM182-LEU2) was performed as previously described (8). In brief, 30 AUs of yeast cells were collected from exponentially growing cultures and resuspended in 1 ml of SCD lacking uracil. Cultures were treated with 50 μCi of [5,6-^{3}H]-Uracil (American Radiolabelled Chemical Inc.,40 Ci*mmol^{-1}) for 5 min and then chased with 5 mM non-radioactive uracil for 0, 5, 10, and 20 min. Aliquots of 5 AU_{600nm} of cells were collected at each time point and immediately snap-frozen in liquid nitrogen. Total RNA was extracted using acidic-phenol and RNA samples were separated on denaturing (formaldehyde), MOPS agarose and (urea), TBE polyacrylamide gels. RNAs were detected using a phosphorimager.

Sucrose gradient analysis.

Sucrose gradient analysis of polysomes was performed as described (9, 10). Sucrose gradient analysis of individual subunits was performed as described (11). In brief, sucrose gradients (10%-40%) were prepared in Beckman tubes (#331374, 14x95 mm Polyallomer) using a Gradient Master 107 IP (Biocomp), program SW40 LONG SUCR 10-40. In both cases, 15 AU of clarified cell extract was loaded, and gradients were centrifuged at 39,000 rpm for 3 hours at 4°C. After centrifugation, fractionation was conducted in Bio-Rad BioLogic LP fractionator while monitoring the absorbance at 260 nm.

Supplementary references

1. Kater,L., Thoms,M., Barrio-Garcia,C., Cheng,J., Ismail,S., Ahmed,Y.L., Bange,G., Kressler,D., Berninghausen,O., Sinning,I., *et al.* (2017) Visualizing the Assembly Pathway of Nucleolar Pre-60S Ribosomes. *Cell*, **171**, 1599-1610.e14.

2. Puig,O., Caspary,F., Rigaut,G., Rutz,B., Bouveret,E., Bragado-Nilsson,E., Wilm,M. and Séraphin,B. (2001) The tandem affinity purification (TAP) method: a general procedure of protein complex purification. *Methods*, **24**, 218–229.

3. Janke,C., Magiera,M.M., Rathfelder,N., Taxis,C., Reber,S., Maekawa,H., Moreno-Borchart,A., Doenges,G., Schwob,E., Schiebel,E., *et al.* (2004) A versatile toolbox for PCR-based tagging of yeast genes: new fluorescent proteins, more markers and promoter substitution cassettes. *Yeast*, **21**, 947–962.

4. Garí,E., Piedrafita,L., Aldea,M. and Herrero,E. (1997) A set of vectors with a tetracycline-regulatable promoter system for modulated gene expression in Saccharomyces cerevisiae. *Yeast*, **13**, 837–848.

5. Bellí,G., Garí,E., Aldea,M. and Herrero,E. (1998) Functional analysis of yeast essential genes using a promoter-substitution cassette and the tetracycline-regulatable dual expression system. *Yeast*, **14**, 1127–1138.

6. Longtine,M.S., McKenzie,A.,3rd, Demarini,D.J., Shah,N.G., Wach,A., Brachat,A., Philippsen,P. and Pringle,J.R. (1998) Additional modules for versatile and economical PCR-based gene deletion and modification in Saccharomyces cerevisiae. *Yeast*, **14**, 953–961.

7. Boissier,F., Schmidt,C.M., Linnemann,J., Fribourg,S. and Perez-Fernandez,J. (2017) Pwp2 mediates UTP-B assembly via two structurally independent domains. *Sci Rep*, **7**, 3169.

8. Kressler,D., de la Cruz,J., Rojo,M. and Linder,P. (1998) Dbp6p is an essential putative ATP-dependent RNA helicase required for 60S-ribosomal-subunit assembly in Saccharomyces cerevisiae. *Mol. Cell. Biol.*, **18**, 1855–1865.

9. Dosil,M. and Bustelo,X.R. (2004) Functional characterization of Pwp2, a WD family protein essential for the assembly of the 90 S pre-ribosomal particle. *J. Biol. Chem.*, **279**, 37385–37397.

10. Pérez-Fernández,J., Román,A., De Las Rivas,J., Bustelo,X.R. and Dosil,M. (2007) The 90S preribosome is a multimodular structure that is assembled through a hierarchical mechanism. *Mol. Cell. Biol.*, **27**, 5414–5429.

11. Foiani,M., Cigan,A.M., Paddon,C.J., Harashima,S. and Hinnebusch,A.G. (1991) GCD2, a translational repressor of the GCN4 gene, has a general function in the initiation of protein synthesis in Saccharomyces cerevisiae. *Mol. Cell. Biol.*, **11**, 3203–3216.

Suppl. Fig. S1

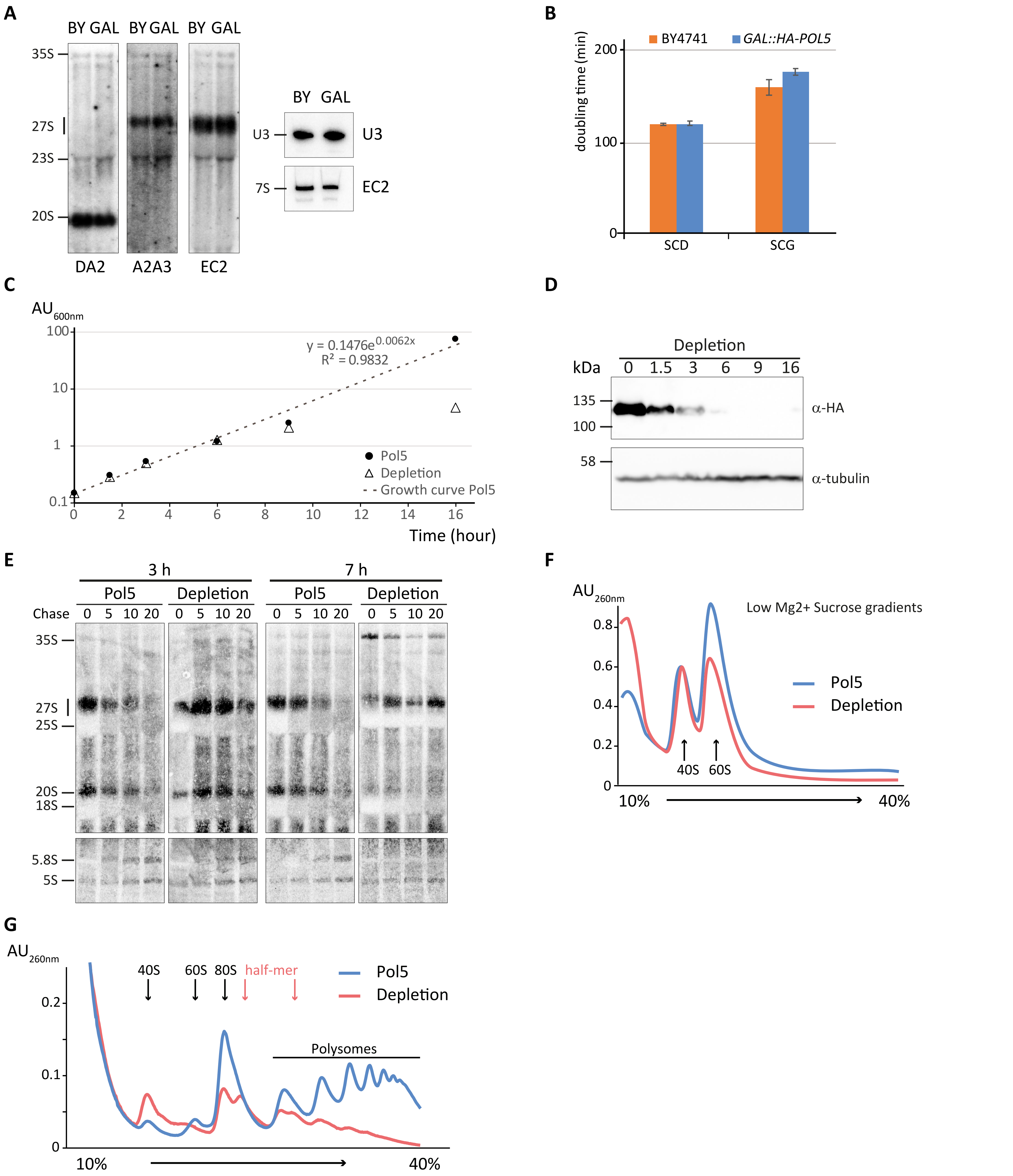
A
BY GAL BY GAL BY GAL
35S
27S
23S
20S
DA2
A2A3
EC2
BY GAL
U3
U3
7S
EC2
B
doubling time (min)
BY4741
GAL::HA-POL5
200
100
0
SCD
SCG
C
AU 600nm
100
10
1
0.1
y = 0.1476e^0.0062x
R² = 0.9832
Pol5
Depletion
Growth curve Pol5
0 2 4 6 8 10 12 14 16
Time (hour)
D
Depletion
kDa
0 1.5 3 6 9 16
135
100
58
α-HA
α-tubulin
E
3 h
7 h
Pol5
Depletion
Pol5
Depletion
Chase
0 5 10 20 0 5 10 20 0 5 10 20 0 5 10 20
35S
27S
25S
20S
18S
5.8S
5S
F
AU 260nm
Low Mg2+ Sucrose gradients
0.8
0.6
0.4
0.2
0
Pol5
Depletion
40S
60S
10%
40%
G
AU 260nm
40S
60S
80S
half-mer
Pol5
Depletion
0.2
0.1
0
Polysomes
10%
40%

Suppl. Fig. S2

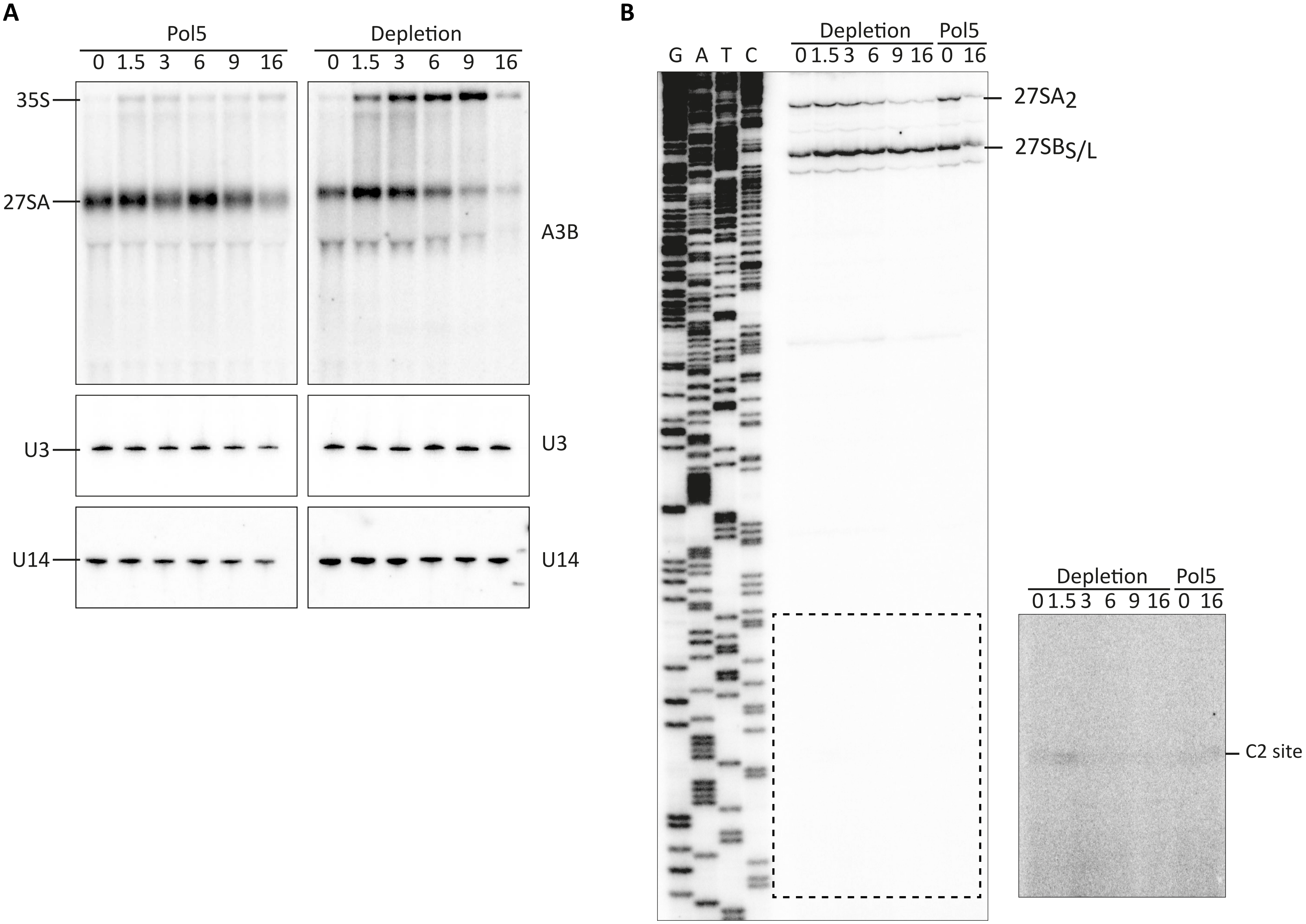
A
Pol5
0 1.5 3 6 9 16
Depletion
0 1.5 3 6 9 16
35S
27SA
A3B
U3
U3
U14
U14
B
G A T C
Depletion
0 1.5 3 6 9 16
Pol5
0 16
27SA2
27SBS/L
Depletion
0 1.5 3 6 9 16
Pol5
0 16
C2 site

Suppl. Fig. S3

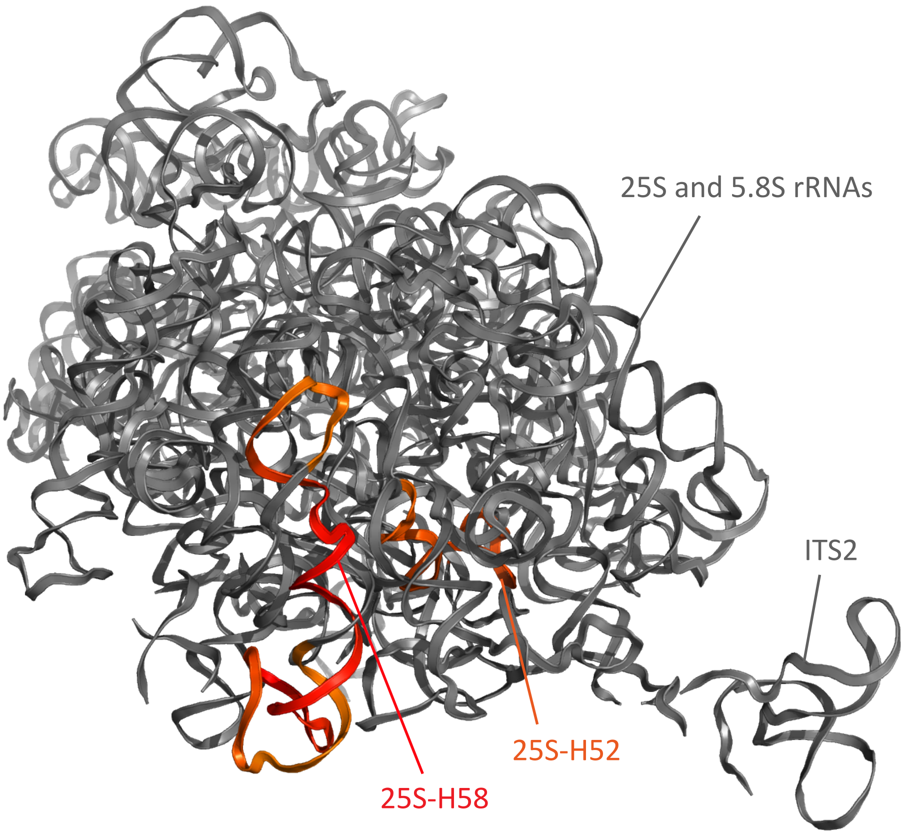

Suppl. Fig. S4

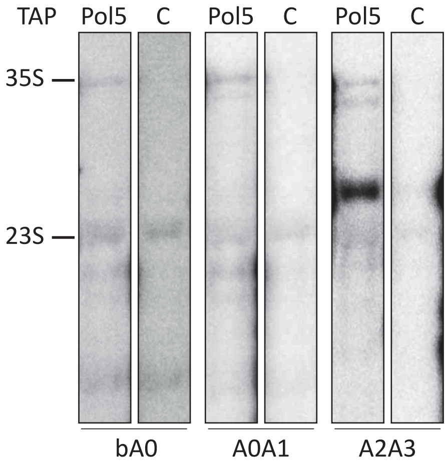

Suppl. Fig. S5

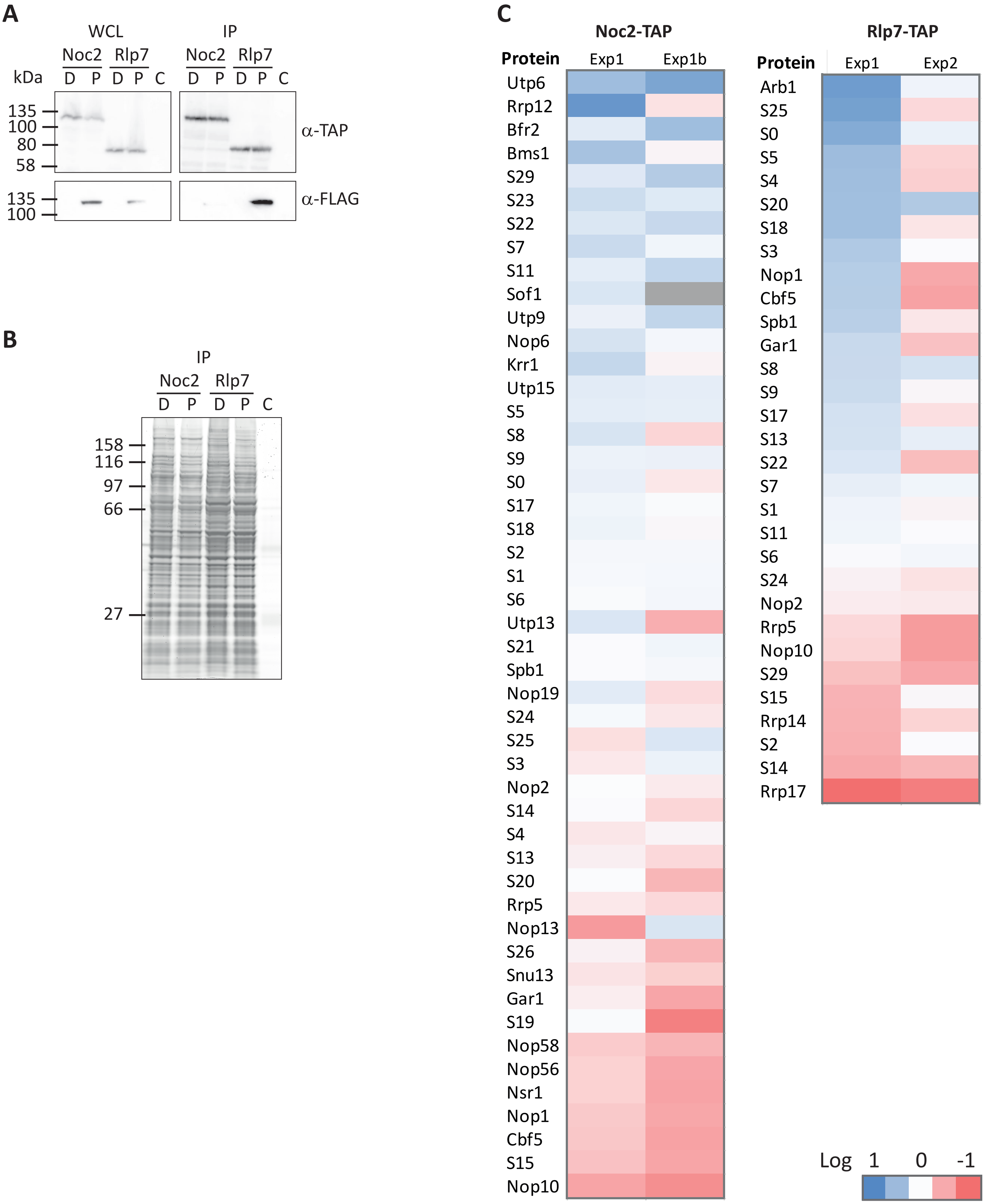
A
WCL
IP
Noc2
Rlp7
kDa
D P D P C
135
100
80
58
α-TAP
α-FLAG
B
IP
Noc2
Rlp7
D P D P C
158
116
97
66
27
C
Noc2-TAP
Protein
Exp1
Exp1b
Utp6
Rrp12
Bfr2
Bms1
S29
S23
S22
S7
S11
Sof1
Utp9
Nop6
Krr1
Utp15
S5
S8
S9
S0
S17
S18
S2
S1
S6
Utp13
S21
Spb1
Nop19
S24
S25
S3
Nop2
S14
S4
S13
S20
Rrp5
Nop13
S26
Snu13
Gar1
S19
Nop58
Nop56
Nsr1
Nop1
Cbf5
S15
Nop10
Rlp7-TAP
Protein
Exp1
Exp2
Arb1
S25
S0
S5
S4
S20
S18
S3
Nop1
Cbf5
Spb1
Gar1
S8
S9
S17
S13
S22
S7
S1
S11
S6
S24
Nop2
Rrp5
Nop10
S29
S15
Rrp14
S2
S14
Rrp17
Log 1 0 -1
